# Successful Use of a 5G-Based Robot-Assisted Remote Ultrasound System in a Care Center for Disabled Patients in Rural China

**DOI:** 10.3389/fpubh.2022.915071

**Published:** 2022-07-18

**Authors:** Hui-hui Chai, Rui-zhong Ye, Lin-fei Xiong, Zi-ning Xu, Xuan Chen, Li-juan Xu, Xin Hu, Lian-feng Jiang, Cheng-zhong Peng

**Affiliations:** ^1^Department of Medical Ultrasound, Shanghai Tenth People' Hospital, Tongji University School of Medicine, Shanghai, China; ^2^Emergency and Critical Care Center, Department of Ultrasound Medicine, Zhejiang Provincial People's Hospital (Affiliated People's Hospital, Hangzhou Medical College), Hangzhou, China; ^3^Department of Engineering, BGI Life Science Research Institution, Shenzhen, China; ^4^Department of General Practice, Yuanshu Disabled Care Center, Huzhou, China; ^5^Ultrasound Research and Education Institute, Clinical Research Center for Interventional Medicine, Tongji University School of Medicine, Shanghai, China; ^6^Shanghai Engineering Research Center of Ultrasound Diagnosis and Treatment, Shanghai, China

**Keywords:** disability, rural health, 5G network, care center, robot-assisted, ultrasonography

## Abstract

**Background:**

Disability has become a global population health challenge. Due to difficulties in self-care or independent living, patients with disability mainly live in community-based care centers or institutions for long-term care. Nonetheless, these settings often lack basic medical resources, such as ultrasonography. Thus, remote ultrasonic robot technology for clinical applications across wide regions is imperative. To date, few experiences of remote diagnostic systems in rural care centers have been reported.

**Objective:**

To assess the feasibility of a fifth-generation cellular technology (5G)-based robot-assisted remote ultrasound system in a care center for disabled patients in rural China.

**Methods:**

Patients underwent remote robot-assisted and bedside ultrasound examinations of the liver, gallbladder, spleen, and kidneys. We compared the diagnostic consistency and differences between the two modalities and evaluated the examination duration, image quality, and safety.

**Results:**

Forty-nine patients were included (21 men; mean age: 61.0 ± 19.0 [range: 19–91] years). Thirty-nine and ten had positive and negative results, respectively; 67 lesions were detected. Comparing the methods, 41 and 8 patients had consistent and inconsistent diagnoses, respectively. The McNemar and kappa values were 0.727 and 0.601, respectively. The mean duration of remote and bedside examinations was 12.2 ± 4.5 (range: 5–26) min and 7.5 ± 1.8 (range: 5–13) min (*p* < 0.001), respectively. The median image score for original images on the patient side and transmitted images on the doctor side was 5 points (interquartile range: [IQR]: 4.7–5.0) and 4.7 points (IQR: 4.5–5.0) (*p* = 0.176), respectively. No obvious complications from the examination were reported.

**Conclusions:**

A 5G-based robot-assisted remote ultrasound system is feasible and has comparable diagnostic efficiency to traditional bedside ultrasound. This system may provide a unique solution for basic ultrasound diagnostic services in primary healthcare settings.

## Introduction

Disability has become a global population health challenge. According to the World Disability Report, approximately 15% of the global population (1 billion people) has various disabilities, and more than 13% of the population with disabilities (85 million people) being in China (1–3). Due to difficulties in self-care or independent living, these patients mainly live in community-based care centers or institutions for long-term care and rehabilitation, rather than being hospitalized (4–6). Care centers for persons with mental disorders or physical disabilities without access to tertiary healthcare services are frequently the primary sites for long-term care and rehabilitation (7–9). However, these settings often lack healthcare resources, including facilities for basic ultrasound examinations (10–12).

Ultrasound imaging is the most easily adaptable diagnostic imaging technology for establishing rapid and non-invasive diagnoses (13). Nonetheless, owing to the lack of skilled sonographers, conducting a traditional on-site ultrasound examination is difficult in several care centers. At care centers for disabled patients, the staff often include mental health professionals, caregivers, and general practitioners only, and there is difficulty in recruiting sonographers (8, 14, 15). Emergencies often force disabled patients to be transferred to nearby hospitals for diagnostic ultrasound examination, increasing the risk of infections and complications related to transportation (15, 16). Advances in telemedicine provide a unique solution to this problem (17–19). With the progressive development of broadband technologies, robotic arm-assisted ultrasound systems improve access to imaging services and close the health equity gap for radiology practices and health systems (19–22).

Over the past 20 years, advances in human-robot interaction systems, master-slave control scheme, and communication technologies have led to the development of remote ultrasonic robot technology for clinical applications across wide geographical regions (16, 23). Furthermore, the availability of off-site medical expertise has become a reality. The initial application of a fifth-generation cellular technology (5G)-based robot-assisted remote ultrasound system during the coronavirus disease (COVID-19) pandemic has achieved encouraging results (22, 24–26). Duan et al. (26) demonstrated that the diagnostic system has considerable value for application in intensive care units. However, to date, none of 5G-based telerobotic ultrasound systems experiences in rural care centers for disabled patients have been reported.

Therefore, we aimed to assess the feasibility of using a 5G-based robot-assisted remote ultrasound system for patients in a rural care center for persons with disabilities in China and to explore a solution for providing basic ultrasound examinations in centers lacking local ultrasound experts and conventional ultrasound devices.

## Materials and Methods

### Patients

The present study involved patients living in the Yuanshu Disabled Care Center, Deqing County, Huzhou, Zhejiang Province, located 35.9 km from the hospital where the 5G-based tele-ultrasound examinations were remotely performed by experts (tele-doctor) in real time, from March to April 2021. All examinations were performed after obtaining written informed consent from each patient or their family and were approved and were approved by the Human Ethics Review Committee of the Zhejiang Provincial People's Hospital.

The inclusion criteria were as follows:

(1) Clinical indications for acute abdomen, such as vomiting, diarrhea, constipation, or flatulence;
(2) Chronic abdominal distention, discomfort, or abdomen mass;
(3) No abdominal ultrasound performed in the previous year.

The exclusion criteria were as follows:

(1) Refusal or inability to cooperate with the ultrasound examination;
(2) Abdomen covered with dressings.

### Instruments

**Figure 1:**
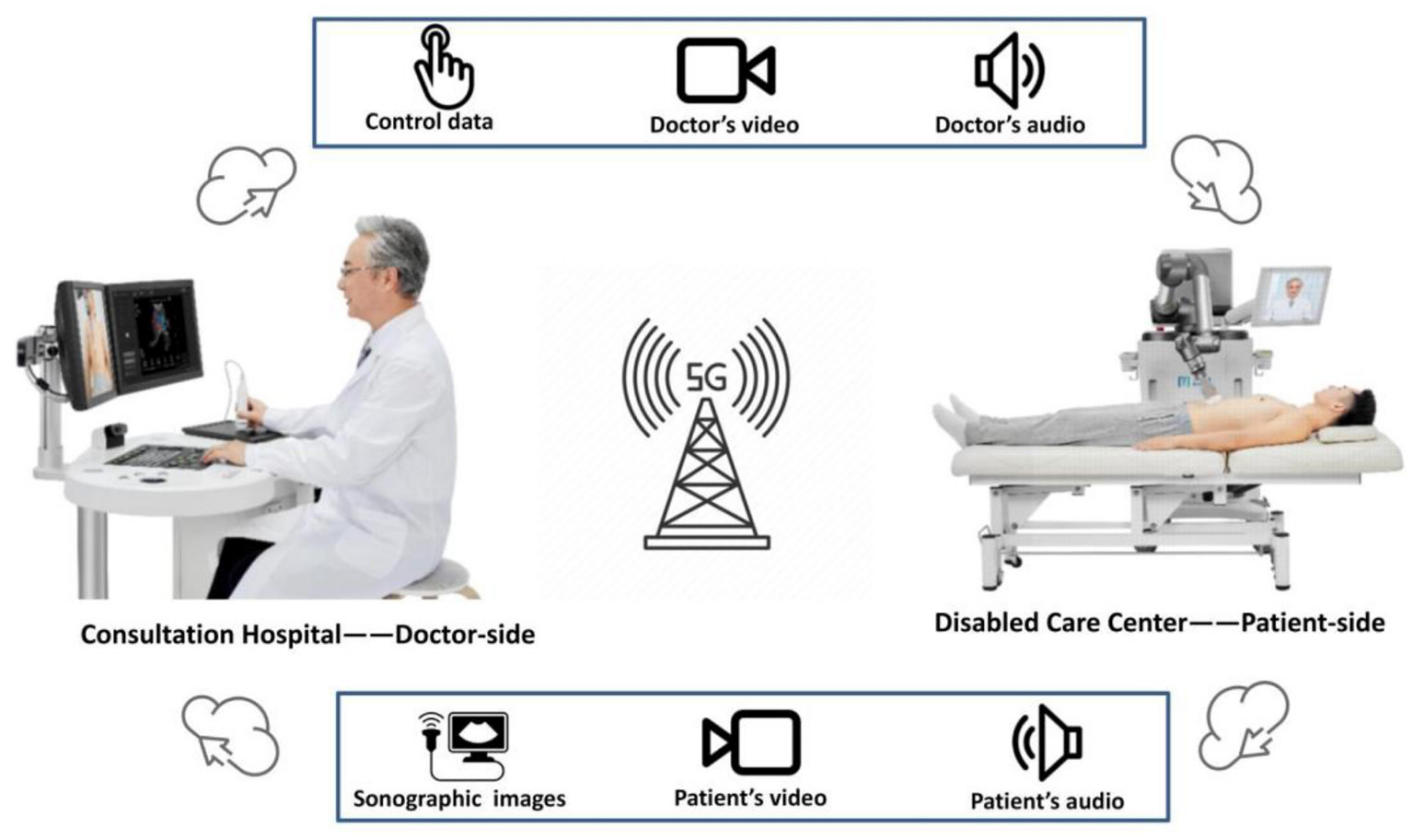
The 5G-based robot-assisted remote ultrasound (MGIUS-R3) comprises two parts: the doctor-side subsystem and the patient-side subsystem. The control data, doctor's operational video, and voice are sent in real time to the patient side through the 5G network. The sonographic images, video of robotic arm manipulation, and patient's audiovisual signal are sent in real time to the doctor side.

A 5G-based robot-assisted remote ultrasound system (MGIUS-R3; Wisonic Medical Technology Co., Ltd., Shenzhen, China) was used for the tele-examinations. The MGIUS-R3 consists of two parts-namely, the doctor-side and the patient-side subsystems. The two systems are connected *via* a speed, low-latency, and large-bandwidth 5G network with a downlink rate of 930 Mbps and an uplink rate of 132 Mbps. The delay in the examination process was <200 ms. The doctor-side subsystem consists of a robot-control console, real-time image display system, ultrasound control panel, and audiovisual communication system. The robot-control console comprised a robotic ultrasound probe, position sensor, and pressure sensor. The robotic ultrasound probe has a posture sensor and “UP button”. The posture sensor managed three degrees of freedoms (DOFs) for rotation, the position sensor managed two DOFs for the movement on the horizontal plane, the “UP button” and pressure sensor managed one DOF for the up and down movement (27). The tele-doctor can manipulate the robot-control console to maneuver a remote robotic arm. The ultrasound parameters including the time gain compensation, focal position, dynamic range, and mechanical index can be adjusted real-time by the tele-doctor *via* the ultrasound control panel. Audiovisual communication system enables synchronous communication between the tele-doctor and on-site assistant or patients. The patient-side subsystem consisted of an ultrasound imaging system, a six-DOF robotic arm, a precise contact force control system, and a audiovisual communication system. The robotic arm (collaborative robot UR5, Universal Robots, Odense, Denmark) was equipped with a convex array probe (frequency: 2.5–5 MHz). Thus, six dimensions of data (three-dimensional rotation, two-dimensional plane and one-dimensional force control) can be collected (22, 26). The robotic arm also contained a high-precision six-dimensional force sensor at the tip to obtain real-time force feedback information when the probe interacted with human soft tissue. The force control and force protection algorithm can ensure the contact force within the set value. For abdomen examination, the vertical protection force of 3–40 N and the horizontal protection force of 20 N could be set, which could ensure the smooth movement of the probe and output stable ultrasound images and protect the patient (26, 27). The screen interface of ultrasound imaging system (Clover 60; Wisonic Medical Technology) is captured by a video capture card and transferred to the doctor side *via* the Internet; the control signal for the ultrasound main unit is captured through the control panel and sent to the main unit following the control protocol. Camera and voice pickup are also included in each subsystem. Through audio–video transmission technology, remote audio-video communication can also be achieved (Figure 1).

**Figure 2:**
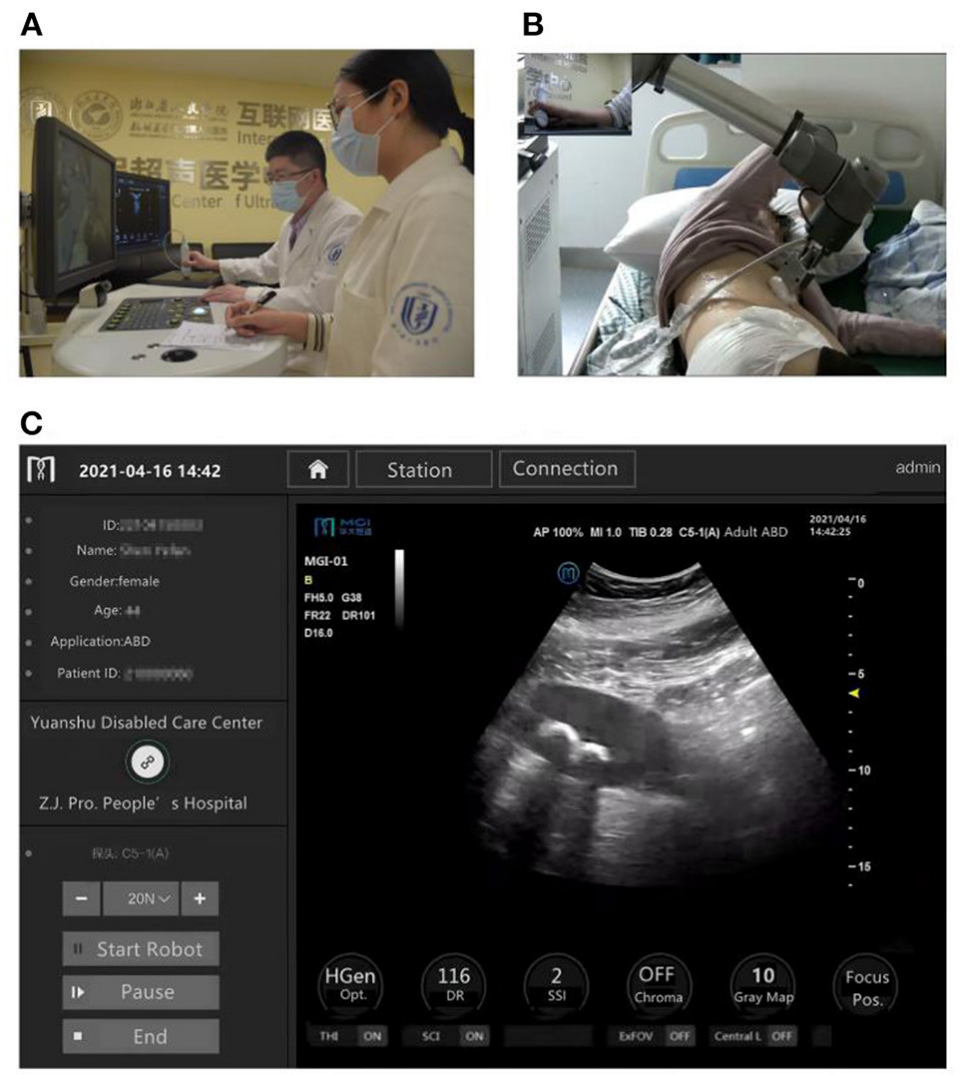
The 5G-based robot-assisted remote ultrasound used for patients in a rural care center for persons with disabilities in China. **(A)** The doctor-side operational scenario in Hangzhou. **(B)** The patient-side operational scenario in Deqing, 35.9 kilometers away from Hangzhou. **(C)** Ultrasound images captured from the patient-side subsystem were sent to the doctor-side subsystem in real time.

In this study, the doctor-side subsystem was located in the Zhejiang Provincial People's Hospital at Hangzhou, and the patient-side subsystem in the Yuanshu Disabled Care Center at Deqing. An expert sonographer remotely operated the robot-control console in the doctor-side subsystem, while the robotic arm of the patient-side subsystem followed the motion instructions transmitted from the doctor side. Then, the real-time sonographic images were sent to the radiologist for evaluation and diagnosis (Figure 2).

A portable ultrasonic diagnostic apparatus (Clover 60; Wisonic Medical Technology) was also used for the bedside examinations.

### Study Design

**Figure 3:**
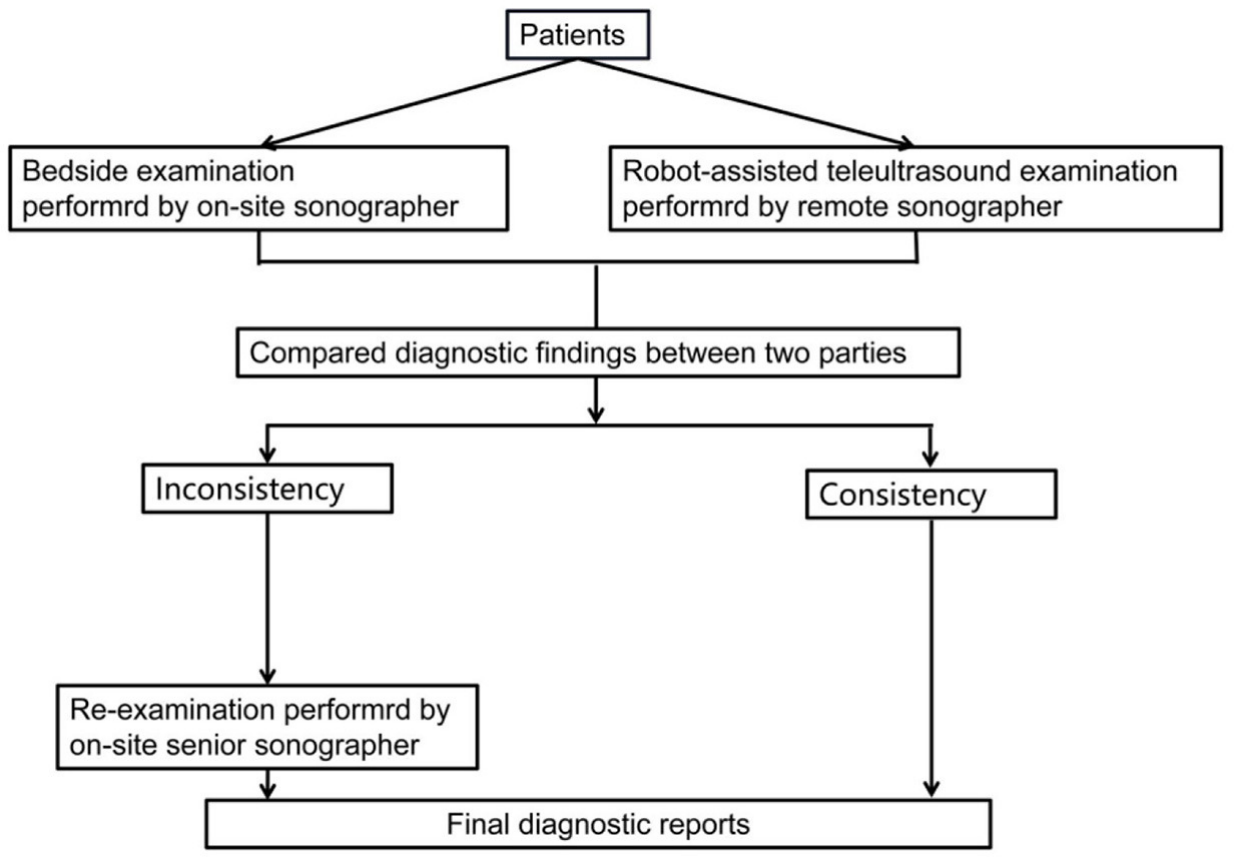
Design of the ultrasound examinations in this study.

The same patients were examined by two independent sonographers using 5G-based robot-assisted remote ultrasound and with standard bedside ultrasound. These sonographers had undergone standardized ultrasound robot manipulation training and had a minimum of 5 years of experience in abdominal ultrasound. Each patient underwent ultrasound examination of the liver, gallbladder, spleen, and kidneys. The ultrasonic findings were classified as positive (a lesion was identified) or negative. If the two examinations were consistent, the results were considered as the final ultrasound diagnosis; otherwise, the patient was re-examined by an on-site senior sonographer (with a minimum of 20 years of abdominal ultrasound experience) to obtain a final diagnosis (Figure 3).

Following the completion of 5G-based robot-assisted remote ultrasound examination, the patients were evaluated to determine the presence of complications related to the examination, including pain, skin lesions, swelling, bleeding, and crush injuries.

Additionally, three independent ultrasonography experts were invited to evaluate the original images on the patient side as well as the images transmitted to the doctor side. The quality of images were evaluated using an internationally defined 5-level absolute evaluation scale 5 points: no image quality deterioration, very good; 4 points: visible image quality changes with unhindered viewing, good; 3 points: image quality deterioration that slightly hindered viewing, fair; 2 points: hindered viewing, poor; 1 point: severely hindered viewing, very poor (26).

### Data Analysis

Statistical analysis was performed using SPSS software version 26.0 (IBM Corp., Armonk, NY, USA). Continuous variables are presented as mean ± standard deviation for normal distribution or as median and interquartile range (IQR) for skewed distribution. Categorical variables are expressed as percentages of the total. Paired-sample Student's *t*-test or Mann–Whitney U-test were used to compare the data between the groups. Cohen's kappa consistency test and McNemar's test were used to evaluate the diagnostic differences and consistency between remote and bedside positive diagnoses. The significance level was set at *p* < 0.05. The kappa values were interpreted as follows: <0.20, poor; 0.21–0.40, fair; 0.41–0.60, moderate; 0.61–0.80, good; and 0.81–1.00, very good (28).

## Results

Fifty-four patients underwent abdominal ultrasound examinations of the liver, gallbladder, spleen, and kidneys. After the 5G-based robot-assisted remote ultrasound examination, no complications related to the study were found. Five non-diagnosed patients were excluded because of severe intestinal gas interference. A total of 49 patients (21 men and 28 women; mean age 61.0 ± 19.0 [range 19–91] years) were included in the analysis. Table 1 displays the demographic and clinical characteristics of the included patients. Of these patients, 53.1% (26/49) had various chronic disorders, 63.3% (31/49) had a mental disability, and 38.8% (19/49) had a physical disability.

**Table 1:** Demographic and clinical characteristics of the patients included in this study.

**Patient information**	***N***
**Clinical characteristics**	
**Age**, ***y***	
<60	26
60–79	12
≥80	11
**Sex**	
Male	21
Female	28
**Indication of abdominal ultrasonography, number**	
Acute abdomen	5
Chronic abdominal distention	11
Chronic abdominal discomfort	13
Abdomen mass	1
Over 1 year since last abdominal ultrasound	19
**Primary disease, number**	
Alzheimer's disease	9
Organic psychosis	1
Depression	5
Epilepsy	1
Schizophrenia	18
Mental retardation	2
Down's syndrome	1
Subacute degeneration of spinal cord	1
Dysopia	4
Sequelae of cerebral infarction	9
Poliomyelitis	1
Fracture	2
Shoulder-hand syndrome	1
Parkinson-plus syndrome	1
Hypertension	23
Diabetes	11
Chronic kidney diseases	1
Chronic incomplete intestinal obstruction	1

**Figure 4:**
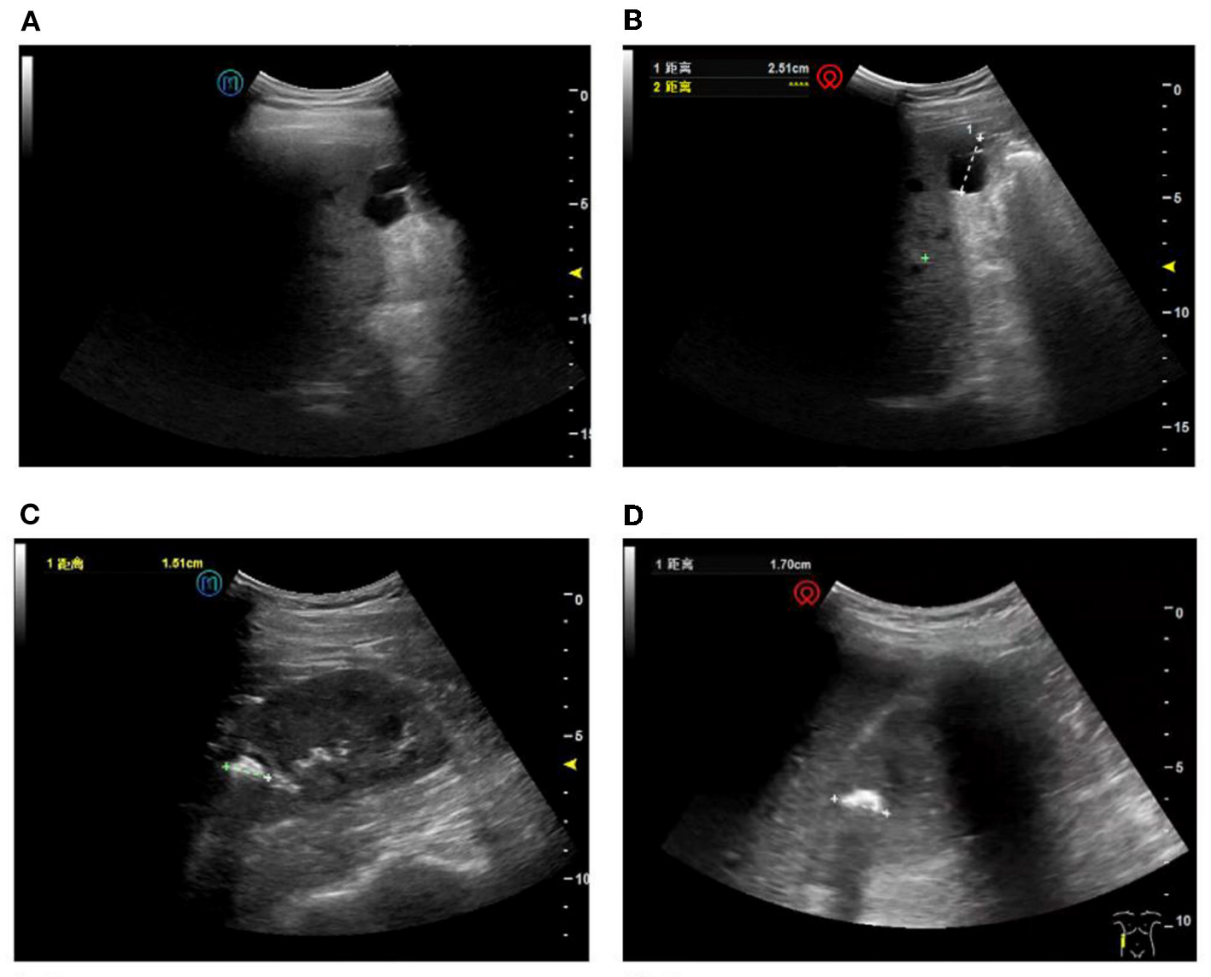
Comparison of the results of the two ultrasound examinations. **(A)** Remote ultrasound image showing a liver cyst. **(B)** Confirmation of the liver cyst using bedside ultrasound. **(C)** Remote ultrasound image showing a right kidney stone. **(D)** Confirmation of the lesion using bedside ultrasound.

Among the 49 patients, 39 and 10 had positive negative ultrasound results. As shown in Table 2, 41 patients had consistent diagnoses between bedside and remote ultrasound, while eight had inconsistent diagnoses. The inconsistent diagnoses included five positives on bedside ultrasound and negative on the remote one, as well as three with opposite results. The McNemar value associated with the two methods was 0.727, and the kappa value was 0.601(*p* < 0.001), indicating that the overall diagnosis results were similar, and there was no significant difference in the diagnosis level between the two methods. Figure 4 presents a comparison of the results of both ultrasound examinations. Overall, 67 lesions were detected in the 39 positive patients (Table 3). The remote ultrasound detected 62 out of 67 lesions (92.5%); the five missed lesions (7.5%) were two left kidney stones, one gallstone, one gallbladder polyp, and one liver cyst. The bedside ultrasound detected 64 out of 67 lesions (95.5%); the three missed lesions (4.5%) were one gallstone, one left kidney cyst, and one right kidney cyst.

**Table 2:** Comparison of the results of the 5G robot-assisted remote and bedside ultrasound examinations.

	**Bedside ultrasound**		**Total**
	**Negative diagnosis**	**Positive diagnosis**	
**Remote ultrasound**			
Negative diagnosis	10	5	15
Positive diagnosis	3	31	34
Total	13	36	49

**Table 3:** Comparison between bedside and remote ultrasound examinations.

**Positive diagnosis**	**Number**	**Inconsistent remote-bedside**	**Kappa value**	***p*-value**	**Strength of agreement**	**McNemar value**
Liver cyst	7	1	0.911	*p < * 0.001	Very good	1
Fatty liver	14	0	1	*p < * 0.001	Very good	1
Hyperechogenic liver	3	0	1	*p < * 0.001	Very good	1
Enlarged gallbladder	1	0	1	*p < * 0.001	Very good	1
Intrahepatic calcification	2	0	1	*p < * 0.001	Good	1
Hepatic hemangioma	1	0	1	*p < * 0.001	Very good	1
Hepatocellular carcinoma	1	0	1	*p < * 0.001	Very good	1
Gallbladder polyps	2	1	0.657	*P < * 0.001	Good	1
Gallstone	5	2	0.728	*p < * 0.001	Good	1
Left kidney cyst	9	1	0.929	*p < * 0.001	Very good	1
Right kidney cyst	6	1	0.898	*p < * 0.001	Very good	1
Left kidney stone	6	2	0.778	*p < * 0.001	Very good	0.5
Right kidney stone	8	0	1	*p < * 0.001	Very good	1
Enlarged spleen	1	0	1	*p < * 0.001	Very good	1
Spleen calcification	1	0	1	*p < * 0.001	Very good	1

The mean remote examination time was 12.2 ± 4.5 (range: 5–26) min, whereas the mean bedside time was 7.5 ± 1.8 (range: 5–13) min, with the difference being significant (*p* < 0.001).

**Figure 5:**
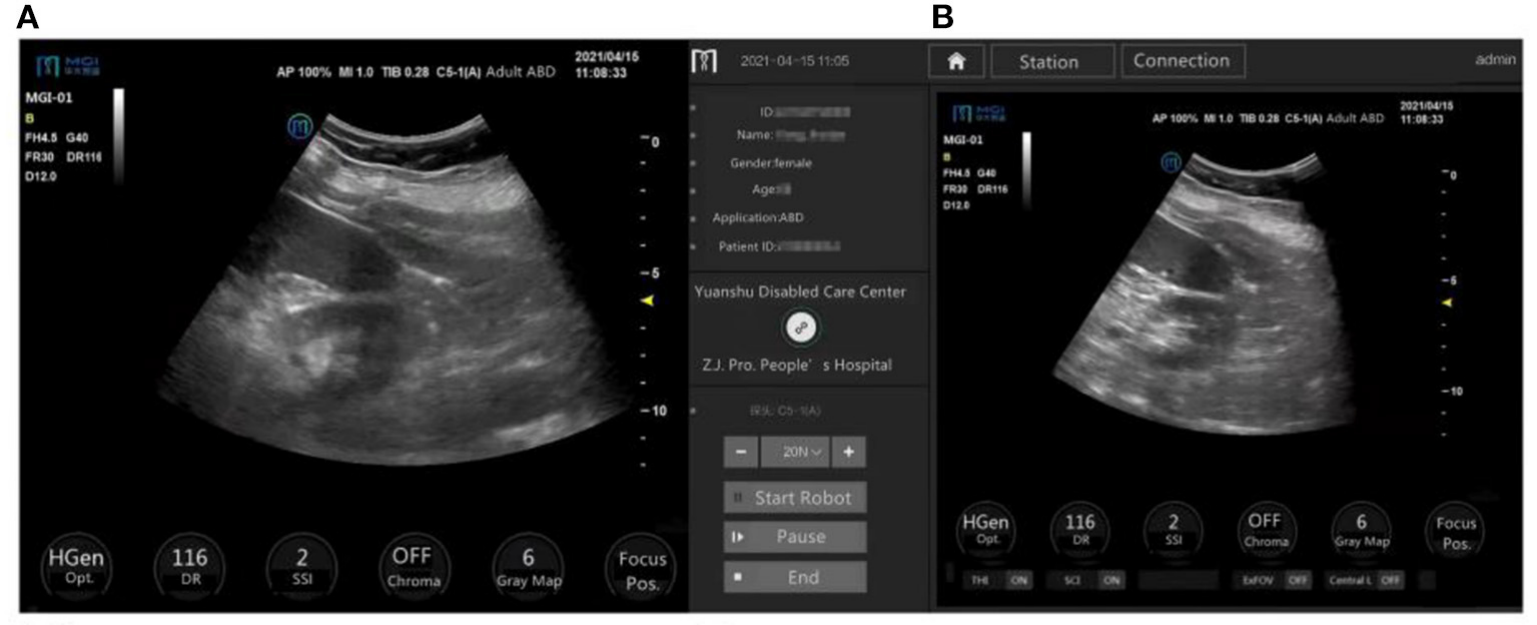
Ultrasound images on the patient side (original images) and doctor side (transmitted images) of the same organs in the same patient. The ultrasound image of the left kidney and the inferior pole of the spleen, displayed on the patient-side, scored 4.3 points on average **(A)**, and on the doctor side scored 4.7 points on average **(B)** according to three independent experts.

Figure 5 shows a comparison of a representative original image at the patient side and a transmitted image at the doctor side.

In the patient-side and doctor-side subsystems, the images with a score of 5 were 72.1 and 68.7%, respectively. The median image scores were 5 points (IQR: 4.7–5.0) and 4.7 points (IQR: 4.5–5.0), respectively (*p* = 0.176), with no significant difference in the image quality.

## Discussion

This study compared a 5G-based robot-assisted remote ultrasound with a traditional bedside ultrasound in 49 patients at a care center for persons with disabilities, located 35.9 km away from the main hospital. Overall, 7.5% and 4.5% of the lesions were undiagnosed by remote ultrasound and bedside ultrasound, respectively. The overall diagnostic results were similar between the remote and bedside ultrasound systems, with no significant differences. This study illustrates that a 5G-based robot-assisted remote ultrasound system may be an effective alternative to traditional bedside ultrasound for abdominal lesion evaluation. In this study, the examination time was longer with remote ultrasound than with bedside ultrasound. This may be related to the difficulty of most patients to completely follow the ultrasound operator's remote command to change the position because of movement disorders (e.g., cerebral infarction sequelae) or mental illness (e.g., schizophrenia). However, the use of 5G-based robot-assisted remote ultrasound system in persons with disabilities decreases the total process time, as compared with routine ultrasound. In particular, patients from remote areas take a longer time to arrive at medical facilities and undergo an ultrasound examination (29). This study suggests that the 5G-based robot-assisted remote ultrasound system will hopefully provide patients at rural care centers with the same diagnostic possibilities as those at tertiary hospitals where experts are instantly available.

With respect to safety, all patients successfully completed the 5G-based robot-assisted remote ultrasound examination. No patient was hurt by the sonographic robotic arm or complained of discomfort during or after the 5G-based robot-assisted remote ultrasound examination. The MGIUS-R3 benefits from the following multiple protection measures for ensuring patient safety (22, 25–27): First, when the robotic arm starts moving, the start prompt is displayed on both terminals. Second, an emergency stop button is installed next to the ultrasound probe socket of the robotic arm on the patient's side. Third, the robotic arm has a default speed of 0.675 m/s for the convex array probe, with parameters that can be changed in real time (i.e., the robotic arm stops moving if the set value exceeds the standard). Fourth, the robotic arm has a force sensor that can control the position and contact force on the patient in real time with pressure-limit settings. Overall, the telerobotic ultrasound system could provide a high degree of safety.

Determining whether or not the ultrasound image quality displayed in the doctor-side subsystem is reliable is crucial (30, 31). Ye et al. (25) investigated the feasibility of a 5G-based robot-assisted remote ultrasound system for the examination of COVID-19 patients, specifically for cardiopulmonary examinations (echocardiography) and evaluation of peripheral lung lesions (lung ultrasound). Ye et al. (25) utilized tele-ultrasound to assess dynamic organs (cardiopulmonary assessment) and revealed that this system is capable of obtaining subtle, complex, and detailed findings. In the current study, we evaluated the received images with respect to gray levels, brightness, and resolution and compared them to original images using a 5-level absolute evaluation scale. Our results indicated that the median scores for the original and transmitted images were comparable (telerobotic ultrasound vs. bedside ultrasound, 5 points [IQR: 4.7–5.0] vs. 4.7 points [IQR: 4.5–5.0]; *p* = 0.176). This finding suggests that there is no perceived degradation in the quality of ultrasound images captured by robot-assisted remote ultrasound systems and that the images can be effectively used for diagnostic purposes.

For the 5G-based robot-assisted remote ultrasound system, it is necessary to ensure high-precision synchronous transmission (32). 5G has the advantages of high speed, greater bandwidth, low latency, and higher reliability (33). In 5G systems, it is thought that 5G download speeds and upload speeds could eventually reach as high as 20 gigabits per second (Gbps) and up to 10 Gbps, respectively (33). The latency is expected to reach a maximum latency of 1 ms with reliability for a packet size of 32 bytes at the user plane (34, 35). The International Telecommunication Union defines three categories of 5G application scenarios—namely, extended mobile broadband, ultra-reliable low-latency communication (URLLC), and massive machine-type communication (33). URLLC can support diverse settings for telemedicine, including telesurgical robots, remote supervision of procedures, integrated intelligent operating rooms, and clinician telepresence (33, 36–39). With network reliability and improvement in latency, realizing the promises of remote ultrasound examination that have been present since the first remote ultrasonic robot system (i.e., the TER system) was reported in 2003 (40). Data streams can more effectively meet the encoding requirements for conveying ultrasound images and videos than the transmission carrying capacity of traditional 4G networks (41). Therefore, no noticeable delay occurred during scanning, and each examination was completed rapidly and almost in real time. In our study, remote experts could fulfill the aims of remote operation and diagnosis. The remote operation and diagnosis were possible due to the 5G network, which provides sufficient transmission speed and reliability in real time across large distances (42, 43).

However, this study has some limitations. First, the 5G-based robot-assisted remote ultrasound system depends on the operator's technical level and requires further improvements. Second, restrictions on patients' examination position and robotic arm's the operating angle occasionally impeded the operator from reaching the regions to be examined. Third, the number of patients enrolled in this study was relatively small, and larger studies are needed in the future.

In conclusion, we demonstrated that the use of a 5G-based robot-assisted remote ultrasound system is feasible in patients with disabilities at a remote care center. Additionally, the 5G-based robot-assisted remote ultrasound system exhibits similar diagnostic efficacy to traditional bedside ultrasound.

## Data Availability Statement

The datasets presented in this article are not readily available because of privacy and ethical restrictions. Requests to access the datasets should be directed to the corresponding author.

## Ethics Statement

The studies involving human participants were reviewed and approved by the Human Ethics Review Committee of the Zhejiang Provincial People's Hospital. We followed relevant guidelines to ensure that this study was voluntary and confidential.

## Author Contributions

C-zP, R-zY, and L-jX designed the study. H-hC, R-zY, C-zP, Z-nX, L-jX, XH, and L-fJ gathered the data. H-hC, R-zY, and L-fX analyzed the data. H-hC, R-zY, C-zP, L-fX, and XC drafted the manuscript. H-hC, R-zY, C-zP, and L-fX revised the manuscript. All authors read and approved the final manuscript.

## Funding

This work was supported by the Zhejiang Medicine Scientific and Technology Project (grant numbers: 2021KY026 and 2022PY003).

## Conflict of Interest

The authors declare that the research was conducted in the absence of any commercial or financial relationships that could be construed as a potential conflict of interest.

## Publisher's Note

All claims expressed in this article are solely those of the authors and do not necessarily represent those of their affiliated organizations, or those of the publisher, the editors and the reviewers. Any product that may be evaluated in this article, or claim that may be made by its manufacturer, is not guaranteed or endorsed by the publisher.
